# A five-year study of the impact of nitrogen addition on methane uptake in alpine grassland

**DOI:** 10.1038/srep32064

**Published:** 2016-08-30

**Authors:** Ping Yue, Kaihui Li, Yanming Gong, Yukun Hu, Anwar Mohammat, Peter Christie, Xuejun Liu

**Affiliations:** 1 State Key Laboratory of Desert and Oasis Ecology, Xinjiang Institute of Ecology and Geography, Chinese Academy of Sciences, Urumqi 830011, China; 2 College of Resources and Environmental Sciences, China Agricultural University, Beijing 100193, China; 3 University of the Chinese Academy of Sciences, Beijing 100039, China

## Abstract

It remains unclear how nitrogen (N) deposition affects soil methane (CH_4_) uptake in semiarid and arid zones. An *in situ* field experiment was conducted from 2010 to 2014 to systematically study the effect of various N application rates (0, 10, 30, and 90 kg N ha^−1^ yr^−1^) on CH_4_ flux in alpine grassland in the Tianshan Mountains. No significant influence of N addition on CH_4_ uptake was found. Initially the CH_4_ uptake rate increased with increasing N application rate by up to 11.5% in 2011 and then there was gradual inhibition by 2014. However, the between-year variability in CH_4_ uptake was very highly significant with average uptake ranging from 52.9 to 106.6 μg C m^−2^ h^−1^ and the rate depended largely on seasonal variability in precipitation and temperature. CH_4_ uptake was positively correlated with soil temperature, air temperature and to a lesser extent with precipitation, and was negatively correlated with soil moisture and NO_3_^−^-N content. The results indicate that between-year variability in CH_4_ uptake was impacted by precipitation and temperature and was not sensitive to elevated N deposition in alpine grassland.

Methane (CH_4_) is the second most important greenhouse gas because it contributes approximately 20% to global warming with a relatively high global warming potential (GWP), 28 times that of CO_2_ on a mono-molecular basis over a 100-year period^1^. The CH_4_ concentration in the atmosphere has increased dynamically from 720 ppb before 1750 to 1803 ppb in 2011 because of large anthropogenic CH_4_ emissions since the industrial revolution^1^,^2^. Soils are an important source and sink of CH_4_ which is controlled largely by the activity of methanotrophs and methanogens^3^. Aerobic soils are the second largest sink of atmospheric CH_4_ because atmospheric CH_4_ is largely consumed by the diffusion of CH_4_ and O_2_ into soils and the processes are limited in the zone of active CH_4_ uptake by both temperature and CH_4_ concentration^4^,^5^. Rates of consumption of CH_4_ are high in surface soils^6^, especially in extensive grasslands which are considered to be major sinks of CH_4_ due to their well-aerated conditions^7^. However, it has been found that N addition reduced CH_4_ uptake by 38% based on a meta-analysis of N addition experiments^8^ and this may be due to changes in the activity of methanotrophs and methanogens resulting from N addition and increased CH_4_ concentrations in the atmosphere.

The impact of N addition on CH_4_ uptake is uncertain and it may increase, decrease or show no effect^9^,^10^,^11^,^12^. In grassland ecosystems CH_4_ uptake is determined by the form and the rate of addition of N and on soil type^13^. There has been considerable debate regarding the effect of rate of N addition on soil CH_4_ uptake. Numerous studies have demonstrated that low N levels increase CH_4_ uptake but higher N levels inhibit its uptake^14^. However, several studies report that NH_4_^+^-N inhibited CH_4_ uptake and NO_3_^−^-N also reduced or increased the CH_4_ sink^14^,^15^ in aerated soil, and this may contribute to soil accumulation of NH_4_^+^-N and NO_3_^−^-N and affect CH_4_ uptake further by changing the activity and composition of the methanotrophic microbial community^16^. In addition, short-term and long-term N addition has been found to stimulate CH_4_ uptake in soil, and inhibitory effects or no effects have also been reported^11^,^15^,^17^. Unfortunately, most studies have focused on temperate regions^9^,^13^,^17^ and to date little is known about the response patterns of CH_4_ flux in the long term and under different N application rates in alpine grassland. It is difficult to accurately assess elevated N deposition impacts on CH_4_ uptake due to trigger changes in soil properties by soil accumulation of NH_4_^+^-N and NO_3_^−^-N.

Bayanbulak grassland is the second-largest grassland in China and is an alpine region with semiarid climatic conditions where little is known about the regulation of CH_4_ cycling under conditions of elevated N deposition. Consequently, the Bayanbulak alpine grassland is an ideal region in which to verify the effects of long-term and elevated N deposition on CH_4_ uptake^18^. We therefore conducted an *in situ* experiment from 2010 to 2014 to investigate variation in CH_4_ flux due to elevated N deposition in Bayanbulak alpine grassland. The main aims of the study were to investigate the response characteristics of the CH_4_ flux with different N application rates and environmental factors, in-season and between-year variability, to quantify the effects of N deposition on CH_4_ uptake, examine the underlying mechanisms and thus to understand the CH_4_ flux response in a semiarid alpine grassland region.

## Materials and Methods

### Site and treatment description

The study was conducted at Bayanbulak Grassland Ecosystem Research Station, located in the southern Tianshan Mountains of Central Asia (42°18′–43°34′N, 82°27′–86°17′E) in Xinjiang Uygur Autonomous Region, west China and administered by the Xinjiang Institute of Ecology and Geography, Chinese Academy of Sciences. The grassland is in the Tianshan Mountains basin at a mean altitude of 2500 m and covers a total area of approximately 23,000 km^2^. The mean annual temperature in the study area is −4.8 °C with mean monthly temperatures ranging from −27.4 °C in January to 11.2 °C in July. The mean annual precipitation is 265.7 mm and is mainly distributed within the growing season from May to August which accounts for 78.1%^18^. The growing season runs from late April to late September. The dominant plant species belong to the Gramineae and include *Stipa purpurea, Festuca ovina*, and *Agropyron cristatum*.

Four N addition treatments were established in April 2010. Nitrogen application rates were set up at 0 (N0), 10 (N10), 30 (N30), and 90 (N90) kg N ha^−1^ yr^−1^. Each treatment was established in four blocks each 4 × 8 m with a 1-m-wide buffer zone. The form of N applied was ammonium nitrate divided into two equal parts and the N was added to the plots in late May and June each year from 2010 to 2014. The NH_4_NO_3_ was weighed, dissolved in 8 L water, and applied to each block using a sprayer to distribute the fertilizer evenly.

### Measurement methods

Soil CH_4_ sorption was measured using a static chamber (50 × 50 × 10 cm) method and gas chromatography (GC). Air samples were taken weekly from 10:00–12:00 (GMT + 8) across the observation period. The air samples were analyzed by GC with a flame ionization detector for CH_4_ (Agilent 7890A, Agilent Technologies, Santa Clara, CA). Soil samples were collected monthly near the static chambers to a depth of 10 cm by auger (3.5 cm diameter) and roots and gravel were removed by passing through a 2-mm sieve. Sieved soil samples were extracted in 0.01 mol L^−1^ CaCl_2_ solution (soil:water 12:100) and the extracts were analyzed for NO_3_^−^-N and NH_4_^+^-N using an Auto-Analyzer 3 (Seal AA3, Bran+Luebbe, Norderstedt, Germany). Soil moisture (S_m_) and soil temperature (T_s_) were measured at 10 cm depth continuously during the five-year study period by an automatic weather station (Campbell Scientific, Logan, UT)^19^.

No winter measurements were made from 2012 to 2014 because of the harsh weather conditions which prevented access to the study area. Winter results from 2010 and 2011 show that the CH_4_ uptake rate was very consistent with a coefficient of variation of 2.20%. The CH_4_ flux in winter from 2012 to 2014 was therefore estimated using the results observed in 2010 and 2011.

### Effects of N addition on CH_4_ uptake

The N addition effect was estimated using the following equation to better quantify the effect of N on the soil CH_4_ flux^20^:


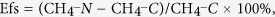


where Efs is the N addition effect on CH_4_ uptake (a positive value indicates that N addition has enhanced CH_4_ uptake and a negative value indicates inhibition of CH_4_ uptake), CH_4_-*N* represents CH_4_ flux from the N addition plots (μg C m^−2^ h^−1^) and CH_4_-*C* denotes the CH_4_ flux from the control plots (μg C m^−2^ h^−1^).

### Statistical analysis

All data are presented as mean and standard error of mean unless otherwise stated. Analysis of variance (ANOVA) and Duncan’s multiple range test (at the 5% level) were used to examine differences in soil temperature, soil moisture, air temperature, precipitation, soil inorganic nitrogen, and CH_4_ flux between the control and N addition plots. In addition, Pearson correlation, stepwise linear regression, three-way ANOVA and linear regressions were used to test the relationships between CH_4_ uptake and soil moisture, soil temperature, precipitation and soil nitrate-N and ammonium-N contents. Curvilinear regression was used to test the relationships between the N addition effect on CH_4_ uptake and soil moisture, soil temperature, and soil available nitrogen. Repeated measures ANOVA was used to examine the effects of year (Y) and nitrogen (N) addition on soil CH_4_ uptake. The Van’t Hoff equation (y = a e^10b^) was used to relate CH_4_ uptake to a change in T_s_ (Q_10_ = e^10b^), soil temperature at 10 cm depth. All statistical analysis was conducted using the SPSS software package version 18.0 (SPSS, Chicago, IL) and differences were considered to be statistically significant at P < 0.05. All figures were drawn using the Sigmaplot version 10.0 software package (SyStat Software Inc., San Jose, CA).

## Results

### Weather conditions and soil properties

The between-year air temperatures and precipitation are shown in Table 1. The air temperature reached its maximum in July. The between-year growing season average air temperature was 8.27 ± 0.34 °C throughout the observation period. Precipitation occurred mainly in June and July (up to 79.1 ± 24.3 mm per month) and especially in July while the average precipitation in May was only 24.0 ± 9.19 mm. The average between-year precipitation during the growing season was 269.3 ± 30.33 mm throughout the observation period. The total precipitation during each growing season was 299.4, 291.2, 246.2, 240.3 and 205.3 mm from 2010 to 2014, respectively. The coefficients of variation (CV) of the results indicate that the precipitation showed large between-year variation (Table 1). Cumulative precipitation showed larger between-year variation (CV 15.1%) than did the air temperature (CV 4.07%). In addition, soil temperature and soil moisture showed significant seasonal variation at 10 cm depth, with a maximum mean monthly soil moisture content in June or July, a minimum in May, and a range of 6.5 to 24.2 g kg^−1^. The maximum soil temperature occurred in July or August with a minimum in May and a range of 3.76 to 12.6 °C during the growing season (Figure S1).

The five-year control and experimental plots show that N addition had no significant influence on soil organic carbon, soil moisture or major plant nutrients (P and K) at different rates of N addition but soil NH_4_^+^-N and NO_3_^−^-N contents increased significantly with increasing N application rate (Table 2). However, due to the high N loss rate of up to 45–52% at our study site^21^, the soil NO_3_^−^-N and NH_4_^+^-N contents did not increase significantly in between-year variation, but the soil NH_4_^+^-N content showed a gradually increasing trend and soil NO^3^_-_-N content showed no significant change in between-year variation (Figure S2). In addition, Figure S3 shows that no significant variation was found in aboveground biomass at the different N application rates but showed large between-year variation. In addition, we found that the interactions of soil moisture, soil temperature, and soil available N significantly increased CH_4_ uptake, except interactions between soil moisture and soil temperature with soil available N (Table S1). Soil moisture and soil temperature therefore significantly impacted the N addition effect on CH_4_ uptake.

### Effect of different N addition rates on soil CH_4_ uptake

**Figure 1:**
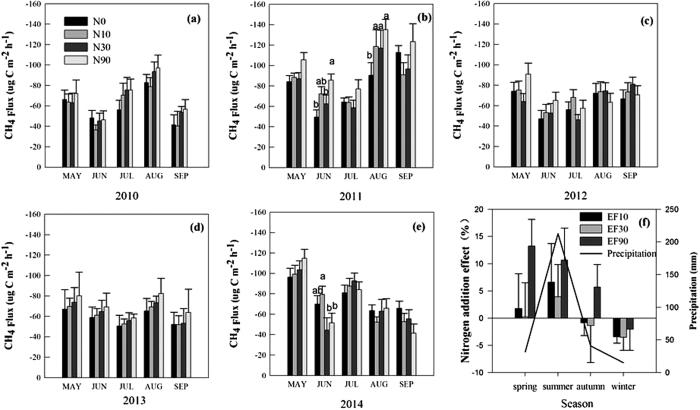
Response of soil CH_4_ uptake to nitrogen deposition and its effect from 2010 to 2014, a positive value representing an increase in soil CH_4_ uptake with increasing N addition (positive effect), a negative value denoting inhibition of CH_4_ uptake (negative effect), and a zero value showing no effect on CH_4_ uptake. N^+^, the amount of N added.

The soil in our alpine grassland was a net C sink in all treatments. The growing season average CH_4_ uptake ranged from 52.9 to 80.2, 53.0 to 90.7, 57.0 to 74.4, and 59.8 to 106.6 μg C m^−2^ h^−1^, respectively, at N application rates N0, N10, N30, and N90 (Table 1) from 2010 to 2014. No significant effect of N addition on CH_4_ uptake was found (Fig. 1) except for a significant increase at N90 in June and August 2011 (Fig. 1b) and inhibition of uptake at N30 and N90 in June 2014 (Fig. 1e). In addition, CH_4_ uptake rate increased with increasing N application rate except at 30 kg N ha^−1^ yr^−1^ and enhanced CH_4_ uptake up to 11.5% in 2011, while the opposite trend occurred in June and September 2014 (Fig. 1e) with a rate of decrease of only 2.9%. Moreover, the effect of N addition on soil CH_4_ uptake decreased gradually and changed with seasonal variability at all N application rates, from an increase in CH_4_ uptake in spring and summer to a gradual inhibition in winter (Fig. 1f).

### Effect of N addition on soil CH_4_ uptake between-year variability

**Figure 2:**
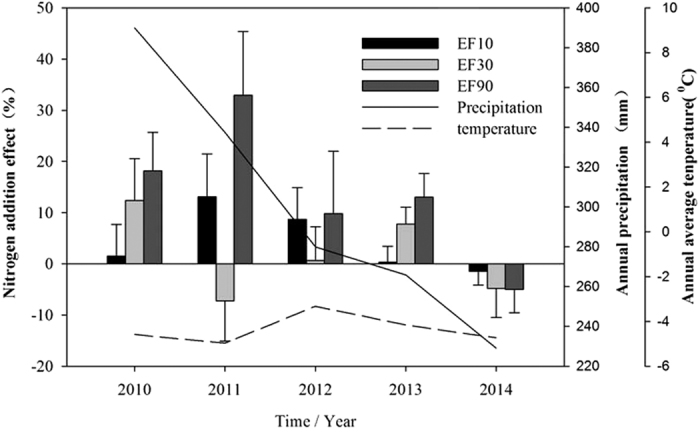
Relationships among N addition effect on CH_4_ uptake (relative to N_0_ treatment), air temperature and precipitation.

No significant influence of N addition on CH_4_ uptake was found (Table 3), though the results of the between-year variability in CH_4_ uptake indicate that the N effect gradually decreased with long-term N addition across the five years of the field experiment. Nitrogen addition enhanced CH_4_ uptake in 2011 by 18.4 to 34.0% (Fig. 2) and this N addition effect diminished over the following year, changing to an inhibitory effect in 2014 (Efs up to −2.88%, Fig. 2). In addition, the contribution of CH_4_ uptake between-year variability was only 5.93% by N addition. However, the between-year variability in CH_4_ uptake was significant (Table 3) and there was large between-year variation in CH_4_ uptake (CV 19.4%) but its variation (CV 17.2%) was consistent with that of precipitation (CV 15.1%) during the growing season, while variation in CH_4_ uptake (CV 2.20%) and air temperature (CV 3.23%) was consistent in the non-growing season, indicating that atmospheric CH_4_ was taken up by the alpine grassland soil and that precipitation and air temperature were important factors during and non-growing season. Non-growing season CH_4_ uptake was lower with a monthly average of 27.3 ± 5.2 μg C m^−2^ h^−1^ due to the snow cover and correspondingly lower temperatures. Methane uptake therefore did not change significantly during the non-growing season (CV 2.20%). Therefore, precipitation and temperature were more important factors affecting CH_4_ uptake between-year variability than was N addition.

## Discussion

### Response of CH_4_ uptake to N addition

No significant effect was observed on soil CH_4_ uptake throughout the observation period by N addition except in June and August 2011 and June 2014 (Fig. 1b,e) when CH_4_ uptake appeared to be promoted at the early stages of N addition and then decreased gradually, but these trends were not significant (Fig. 2) and were most likely due to a higher N loss rate (up to 45–52% of added N) and the effects of soil N saturation^21^. On one hand, N was the main limiting factor and the added inorganic N met the grass demand and increased CH_4_ uptake^22^. However, with the continuous increase in N deposition across China^23^, N in the soil gradually accumulates and reaches saturation or is readily lost to the environment, thus reducing the C/N ratio to give a lack of available carbon and leading to inhibition of microbial activity, including inhibition of CH_4_ uptake. On the other hand, higher litter inputs under N enrichment alleviate microbial C limitation and the activities of methanogenic archaea are enhanced and more CH_4_ is produced. Less CH_4_ is oxidized by methanotrophic bacteria under N enrichment and more CH_4_ is therefore emitted to the atmosphere^8^. In addition, it was previously suggested that microbial biomass declined by 15% on average under N fertilization and declines in the abundance of bacteria and fungi were more evident in long-term studies and higher amounts of N addition^24^, leading to inhibition of uptake. The results indicate that CH_4_ uptake is complicated under elevated N deposition and long-term studies are required. In our study N addition did not significantly impact soil CH_4_ uptake. Initially the CH_4_ uptake rate appeared to increase with increasing N application rate up to 11.5% in 2011 and then gradually changed to inhibition of uptake in 2014 although these apparent changes were not significant. However, inhibition of grassland soil CH_4_ uptake under long-term increase in atmospheric N deposition has been reported by numerous studies^9^,^25^. However, short-term N deposition stimulated CH_4_ uptake in soil and inhibitory or no effects have also been reported^15^,^17^,^26^. In addition, studies on forest ecosystems have shown that the CH_4_ sink gradually switched to a source of CH_4_ with long-term N addition^27^ indicating that the CH_4_ sink in alpine grassland might be weakened or switch to a source with elevated N deposition in the future. Our results might represent a transition period between the N deposition effect of increasing to inhibiting soil CH_4_ uptake. Therefore, long-term observations are needed to elucidate the processes of soil CH_4_ sink or source under conditions of elevated N deposition.

The soils in arid ecosystems play an important role as a sink for atmospheric CH_4_. The trend of the N addition effect is consistent with the seasonal change in precipitation (Fig. 1f), including between-year variability for precipitation and the N addition effect (Fig. 2) and this indicates that interactions between precipitation and elevated N deposition are important for CH_4_ uptake. In addition, CH_4_ uptake was inhibited in winter under elevated N deposition, which is consistent with N addition having a stronger inhibitory effect at lower temperatures^14^. These results indicate that the process of soil CH_4_ uptake is very complicated under elevated N deposition, precipitation and temperature. To summarize, climate change is an important driving factor for CH_4_ oxidation in alpine ecosystems and elevated N deposition, warming and change in rainfall patterns can profoundly affect the soil CH_4_ balance^11^,^28^. Interactions between environmental factors and N addition as they affect CH_4_ uptake need to be considered and short-term studies are inadequate to evaluate the magnitude, spatial distribution and temporal changes involved in the long term.

### Impact of N addition on between-year variability in CH_4_ uptake

The contribution of N addition to CH_4_ uptake was only 5.93% of between-year variability, smaller than the reduction in CH_4_ uptake due to N addition of 38% found by Liu and Greaver^8^ in their meta-analysis of N addition experiments. Furthermore, using repeated measures ANOVA to examine the effects of year (Y) and nitrogen (N) addition on soil CH_4_ uptake (CH_4_), we found that N addition did not significantly impact CH_4_ uptake over the time scale of the study (Table S1). However, the effect of different N application rates on CH_4_ uptake differed. For example, CH_4_ uptake was either promoted or inhibited by N addition (Fig. 2). Furthermore, weak between-year variability was observed in CH_4_ uptake (CV 10.3–23.9%) due to N addition and low N addition rates giving higher variation than the control plots and intermediate N rates giving slightly lower variation than the controls (Table 1). This is consistent with the results of a meta-analysis in which low rates of N addition tended to stimulate CH_4_ uptake while higher N rates were inhibitory or exerted no effect^14^. However, a high N addition rate (90 kg N ha^−1^ yr^−1^) stimulated CH_4_ uptake from 2010 to 2013 and this may indicate a tipping point at about 100 kg N ha^−1^ yr^−1 ^14. Thus, the alpine soil may be N-limited in its consumption of CH_4_ from the atmosphere and there may be interactions between environmental factors and N addition affecting CH_4_ uptake such as low soil temperatures and high precipitation which may lead to low rates of CH_4_ uptake under N addition conditions.

Higher between-year variability in CH_4_ uptake was found in our study, and this may contribute to variability in precipitation and temperature. For instance, strong CH_4_ uptake occurred during the dry season (the maximum CH_4_ uptake rate of 114.2 ± 6.1 μg C m^−2^ h^−1^ in September, Fig. 1b) and the lowest uptake rate averaged only 8.3 ± 1.6 μg C m^−2^ h^−1^ in December (cold season, Table 1). At the between-year scale, the highest cumulative precipitation occurred in 2010 when the CH_4_ uptake was relatively low. Thus, it would appear that precipitation inhibited CH_4_ uptake. Correlation analysis shows that CH_4_ uptake was less closely related to precipitation (CH_4_ uptake = 52.8 + 0.11P, *P* = 0.35, Table 4). However, the lowest CH_4_ uptake occurred in 2013 when precipitation during the growing season was 240.3 mm and the highest CH_4_ uptake occurred in 2011 due to the higher cumulative precipitation in the growing season in 2011 and heavy cumulative snow cover in the non-growing season in 2010 (Table 1). This indicates a time lag affecting soil CH_4_ uptake due to snow cover and an important impact of precipitation. This is consistent with results from the Tibetan Plateau showing that seasonal CH_4_ uptake was controlled mainly by soil moisture rather than air temperature^29^. This may be due to water supply acting as the main limiting factor during the growing season but temperature acting as the major factor in non-growing season. As is well known, CH_4_ uptake decreases during the rainy season (e.g. in July), with a monthly CH_4_ uptake rate of 70.7 ± 5.6 μg C m^−2^ h^−1^ (Fig. 1). This indicates that between-year variability in CH_4_ uptake is dependent on between-year variation in precipitation and air temperature rather than N deposition in alpine regions.

Cumulative precipitation and air temperature can profoundly impact changes in soil moisture and soil temperature and thus further influence the CH_4_ balance in the soil. In our study we found that CH_4_ uptake was positively (*P* < 0.05) correlated with soil temperature and air temperature (CH_4_ uptake = 36.39 + 2.87S_t_, *P* < 0.01, CH_4_ uptake = 50.79 + 1.53A_t_, *P* < 0.01, Table 4) and negatively correlated with soil moisture (CH_4_ uptake = 87.87-0.94S_m_, *P* < 0.01, Table 4), further demonstrating that soil temperature and soil moisture are important factors affecting CH_4_ uptake. Moreover, the higher the soil moisture content the less CH_4_ is taken up and CH_4_ oxidation rates are negatively correlated with soil moisture content and this is consistent with most *in situ* observations^27^,^30^ in which higher CH_4_ uptake has occurred at intermediate soil moisture contents and much higher or very low soil moisture contents have inhibited CH_4_ uptake^29^. The dependence of CH_4_ uptake on soil moisture content can be explained by changes in the activities of methanotrophs and methanogens^31^ and CH_4_ soil-atmosphere exchange tends to reduce the sink of CH_4_ at higher soil moisture contents^32^. We found that N addition was not significantly associated with changes in soil moisture content but positive, negative and neutral results have been reported previously^33^,^34^,^35^, indicating that changes in soil moisture may be induced by N deposition with subsequent influence on CH_4_ uptake. Temperature is also an important factor for CH_4_ uptake by aerated soils, especially in the alpine zone. It has been shown that warming can directly affect CH_4_ oxidation and soil moisture content^2^. Soil temperature in our study area reaches an average of 13 °C in July^19^, a level more suitable for methanogens than methanotrophs because CH_4_ production increases three or four times between 10 and 20 °C and this would reduce CH_4_ uptake in the study area^36^.

The temperature sensitivity (Q_10_) of CH_4_ oxidation was relatively high (Q_10_ 2.31), indicating that soil CH_4_ uptake in the alpine grassland might be more sensitive to warming than in temperate regions. Furthermore, the ambient temperature has increased by 1 °C over the past 50 years and precipitation shows a significant increasing trend in the 1980s and 1990s, and the average annual precipitation shows an increasing trend with a magnitude of 6.8 mm per decade^37^, while warming has decreased soil moisture content and enhanced the potential for oxidation of CH_4_^38^. In addition, soil temperature can also impact potential CH_4_ oxidation rate and CH_4_ solubility^39^. In conclusion, the soil CH_4_ oxidation rate was profoundly impacted by soil moisture and soil temperature, while the changes in soil moisture and soil temperature were controlled by precipitation and air temperature. Significant changes in trends of environmental factors will therefore strongly impact the balance of CH_4_ in the soil, and larger CH_4_ uptake between-year variability depended on changes in precipitation and temperature.

### Mechanism of CH_4_ uptake with inorganic N input

A reduction in soil CH_4_ uptake was observed in response to N addition in 2014 which is consistent with N deposition inhibiting CH_4_ uptake in semi-arid and arid ecosystems^40^,^41^, but N addition increased soil NH_4_^+^-N and NO_3_^−^-N availability, especially at medium and high N addition rates (Table 2). Soil NO_3_^−^-N content significantly decreased soil CH_4_ uptake (CH_4_ uptake = 92.36-0.88NO_3_^−^-N, *P* < 0.01, Table 4) and this is not consistent with stimulation of CH_4_ uptake by soil nitrate at low CH_4_ concentrations because of changes in the CH_4_ oxidizing bacterial community^11^. Methane oxidizers are optimally active at low redox potentials (200 mV) under low NO_3_^−^-N concentrations but CH_4_ uptake is restrained by higher NO_3_^−^-N concentrations^3^ due to increased redox potential and this leads to osmotic effects^24^. Another important factor is that excessive NO_3_^−^-N concentrations may be toxic to CH_4_-oxidizing bacteria^42^. The NH_4_^+^-N content did not significantly impact CH_4_ uptake (Table 4, *P* = 0.20) and this is not consistent with the results of previous studies in which soil CH_4_ uptake was enhanced by increased NH_4_^+^-N availability due to an increase in the number of soil ammonia oxidizing microorganisms^43^,^44^,^45^, and the opposite result is due to the inhibition of CH_4_ oxidation by NH_4_^+^-N due to lack of specificity of CH_4_ monooxygenase^8^. To summarize, soil CH_4_ uptake decreased and this may contribute to the accumlation of soil NO_3_^−^-N content under conditions of elevated N deposition. The CH_4_ uptake rate increased with increasing N application rate (0, 10, 30, 90 kg N ha^−1^ yr^−1^) initially. In our study site soil NO_3_^−^-N content increased soil CH_4_ uptake but soil NH_4_^+^-N content was not significantly influenced due to the high N loss rate at our study site which may result in N addition (NH_4_NO_3_) having no significant impact on CH_4_ uptake rate. We found that insufficient or excessive soil moisture would inhibit the N addition effect on CH_4_ uptake at N90 sites, while soil temperature enhanced the N addition effects on CH_4_ uptake at N90 sites (Figure S3). No significant impact of soil moisture or soil temperature was found at N10 and N30 sites compared with the control plots. On one hand, this indicates that the soil available N content was too low to impact on the N addition effect by soil moisture and soil temperature. On the other hand, the study site soil moisture ranged from 5.21 to 36.3 g kg^−1^ during the observation period, a level to low to impact soil CH_4_ uptake. In addition, the methane uptake rate was significantly increased by interaction effects of soil moisture, soil temperature and soil available N (Table S1) and this is consistent with results showing that the CH_4_ uptake rate depended on interactions of temperature, precipitation and soil available nitrogen in upland areas^14^. This indicates that the N addition effect on the methane uptake rate was influenced by temperature and precipitation.

## Conclusions

No significant effect on CH_4_ uptake was found in alpine grassland of the Tianshan Mountains in a five-year N addition experiment. Initially the CH_4_ uptake rate appeared to increase with increasing N application rate up to 90 kg N ha^−1^ yr^−1^ and enhanced CH_4_ uptake up to 11.5% in 2011, and then gradually switched to inhibition of uptake in 2014. A weak effect on CH_4_ uptake occurred in seasonal variability and large between-year variability, ranging from 52.9 to 106.6 μg C m^−2^ h^−1^, was dependent on variability in precipitation and temperature rather than N deposition.

Precipitation and higher temperature sensitivity (Q_10_) of CH_4_ oxidation were key factors affecting CH_4_ uptake in the alpine semi-arid grassland. Soil CH_4_ uptake was positively correlated with soil temperature and air temperature, but less related to precipitation and negatively to soil moisture and soil NO_3_^−^-N content.

## Additional Information

**How to cite this article**: Yue, P. *et al*. A five-year study of the impact of nitrogen addition on methane uptake in alpine grassland. *Sci. Rep*. **6**, 32064; doi: 10.1038/srep32064 (2016).

## Supplementary Material

Supplementary Information

## Figures and Tables

**Table 1:** Between-year variability in air temperature, cumulative precipitation and CH_4_ uptake rate as affected by N addition treatments within and outside the growing seasons.

Year	Air temp. (°C)	Precip. (mm)	N effects on CH_4_ uptake rate (μg C m^−2^ h^−1^)			
Growing season			N0	N10	N30	N90
2010	7.89	299.4	58.9 ± 8.1	58.0 ± 8.7	66.2 ± 9.5	69.6 ± 7.4
2011	8.40	291.2	80.2 ± 7.4	90.7 ± 9.3	74.4 ± 7.3	106.6 ± 9.7
2012	8.78	246.2	63.3 ± 6.5	68.8 ± 7.6	63.7 ± 6.4	69.5 ± 7.3
2013	8.13	240.3	52.9 ± 8.5	53.0 ± 6.9	57.0 ± 8.2	59.8 ± 8.2
2014	8.16	205.3	75.4 ± 7.8	74.3 ± 9.6	71.7 ± 5.4	71.6 ± 7.6
mean	8.27 ± 0.34	256.5 ± 38.8	66.1 ± 7.6	69.0 ± 8.4	66.6 ± 7.4	75.4 ± 8.0
CV	4.07%	15.1%	17.2%	21.4%	10.3%	23.9%
Outside the growing season						
2010	−16.0	90.5	26.8 ± 5.4	28.5 ± 6.2	28.9 ± 5.4	32.7 ± 7.2
2011	−16.8	46.3	27.7 ± 4.9	30.2 ± 5.7	29.0 ± 5.1	32.2 ± 5.9
Mean	−16.4 ± 0.5	68.4 ± 31.2	27.3 ± 0.6	29.4 ± 1.2	29.0 ± 0.1	32.5 ± 0.35
CV	3.23%	45.7%	2.20%	4.02%	0.24%	1.06%

Air temp. = Air temperature; Precip. = Precipitation.

**Table 2:** N addition impacts on nitrate N, ammonium N and other related soil properties.

Site	NO_3_^—^N (mgkg^−1^)	NH_4_^+^-N (mgkg^−1^)	Soil moisture (g kg^−1^)	TOC (g kg^−1^)	TN (g kg^−1^)	Olsen-P (mgkg^−1^)	Exc.-K (mg kg^−1^)	C/N ratio
N0	13.3 ± 3.1 b	5.4 ± 2.1 b	18.0 ± 4.5	32.7 ± 3.2	3.11 ± 0.28	11.1 ± 1.3	61.9 ± 12.3	10.5
N10	11.8 ± 2.1 b	7.9 ± 2.3 b	16.8 ± 3.1	39.0 ± 5.4	3.35 ± 0.85	12.0 ± 0.8	64.1 ± 14.3	11.6
N30	21.6 ± 2.6 a	10.0 ± 2.4 a	15.5 ± 3.7	38.9 ± 2.4	3.45 ± 0.53	12.2 ± 1.5	65.2 ± 31.8	11.3
N90	23.9 ± 2.1 a	10.5 ± 2.7 a	16.6 ± 2.3	36.3 ± 4.6	3.10 ± 0.71	12.0 ± 1.8	65.8 ± 42.3	11.7

TOC: soil total organic carbon, TN: soil total nitrogen, Exc.-K: Exchangeable K, C/N: ratio of soil organic carbon to soil total nitrogen.

**Table 3:** Nitrogen addition impacts and between-year variability on CH_4_ uptake.

Two-way ANOVA	F	P
N	2.418	0.066
Y	4.631	0.001
N × Y	1.190	0.290

N, nitrogen addition; Y, year.

**Table 4:** Correlations and linear relationships with precipitation, soil moisture, air temperature, soil moisture NH_4_^+^-N content and NO_3_^−^-N content.

Environmental factor	Pearson’s correlation			Y = aX + b			
	r	p	n	a	b	R^2^	p
Precipitation (P)	0.083	0.729	20	0.11	52.84	0.16	0.35
Air temperature (At)	0.346	0.05	32	1.53	50.79	0.35	0.05
Soil moisture (Sm)	−0.282	0.015	73	−0.94	87.87	0.24	0.015
Soil temperature (St)	0.722	0.00	106	2.87	36.39	0.52	<0.0001
NH_4_^+^-N content	0.100	0.20	176	0.41	74.88	0.03	0.20
NO_3_^−^-N content	−0.346	0.00	176	−0.88	92.36	0.12	<0.0001

P, Precipitation, S_m_, Soil moisture, A_t_, Air temperature, S_t_, Soil temperature.
