# Supplementary material for: A five-year study of the impact of nitrogen addition on methane uptake in alpine grassland
Source: Sci Rep. 2016 Aug 30;6:32064. doi: 10.1038/srep32064 (PMC5004186; doi:10.1038/srep32064)
Supplement: Supplementary Information [file srep32064-s1.pdf]

Supplementary Materials

A five-year study of the impact of nitrogen addition on methane uptake in alpine grassland

Ping Yue[1,2,3], Kaihui Li[1,*], Yanming Gong[1], Yukun Hu[1], Anwar Mohammat[1], Peter Christie[2], Xuejun Liu[2,*]

[1]State Key Laboratory of Desert and Oasis Ecology, Xinjiang Institute of Ecology and Geography, Chinese Academy of Sciences, Urumqi 830011, China

[2]College of Resources and Environmental Sciences, China Agricultural University, Beijing 100193, China

[3]University of the Chinese Academy of Sciences, Beijing 100039, China

[*] Correspondence to: Xuejun Liu (liu310@cau.edu.cn) and Kaihui Li (likh@ms.xjb.ac.cn)

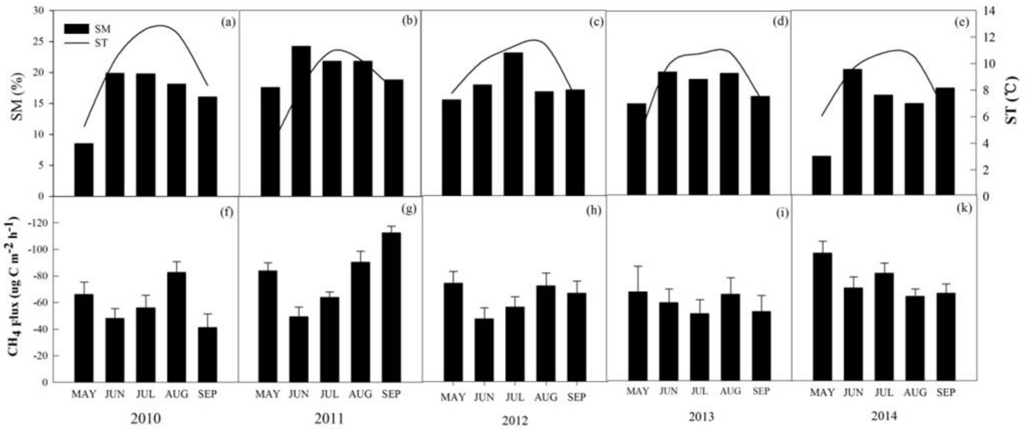

Figure S1 Seasonal variation in soil temperature, soil moisture and soil CH_4 uptake in the alpine grassland. Soil moisture and soil temperature are mean monthly values.

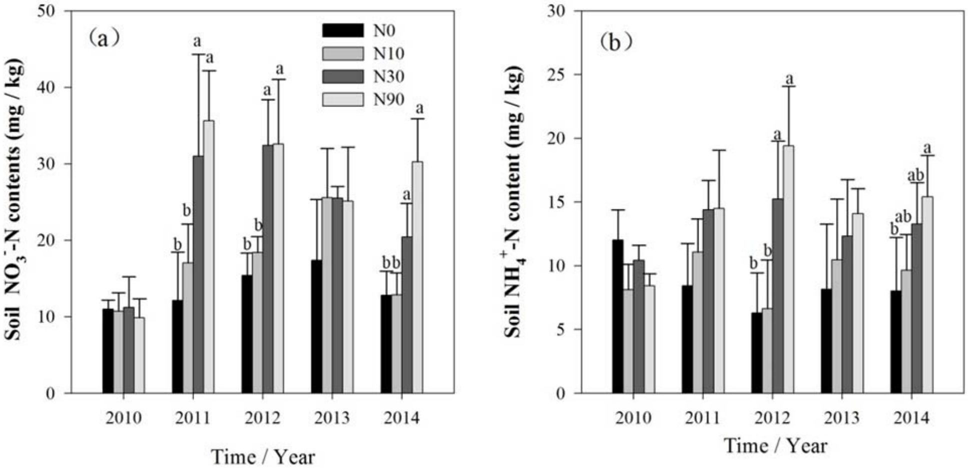

Figure S2 Between-year variation in soil NO_3^--N content and NH_4^+-N content under different nitrogen application rates.

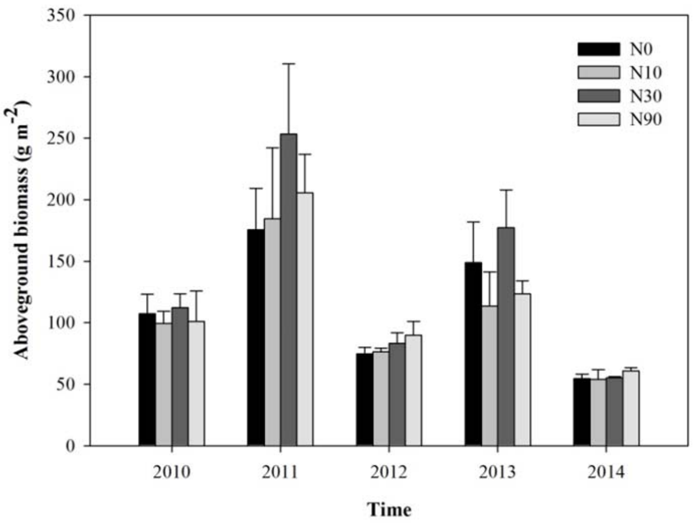

Figure S3 Variation in aboveground biomass under different nitrogen application rates.

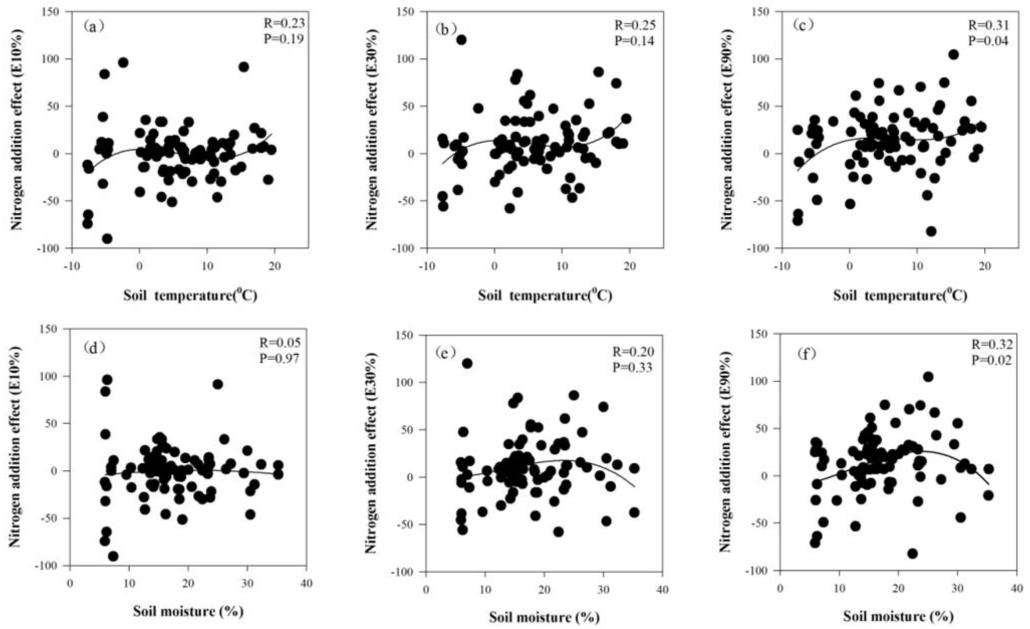

Figure S4 Relationships among N addition effect on CH_4 uptake (relative to N0 treatment) with soil moisture and soil temperature.

Table S1 Results (F and P values) of the repeated measures ANOVA of the effects of year (Y)and nitrogen (N) addition on soil methane uptake (CH_4), and multivariate ANOVA of relationship on CH_4 uptake between soil moisture (SM), soil temperature (ST) and soil available nitrogen content.

	n	F	P
Repeated measures ANOVA			
Y	5	7.605	0.000**
N	5	0.626	0.609
Y×N	5	0.531	0.858
Three way ANOVA			
SM	177	5.539	0.020*
ST	177	36.516	0.000**
N	177	0.473	0.493
SM×ST	177	10.823	0.001**
SM×N	177	0.447	0.504
ST×N	177	0.054	0.815
ST×SM×N	177	4.019	0.047*

Abbreviations: Y, year; N, rate of nitrogen addition; SM, soil moisture, ST, soil temperature . *, ** denote $P < 0.05$ and 0.01 significant levels, respectively.
